# Analysis of copy number variation at *DMBT1* and age-related macular degeneration

**DOI:** 10.1186/s12881-016-0311-5

**Published:** 2016-07-15

**Authors:** Shamik Polley, Valentina Cipriani, Jane C. Khan, Humma Shahid, Anthony T. Moore, John R. W. Yates, Edward J. Hollox

**Affiliations:** Department of Genetics, University of Leicester, Leicester, UK; UCL Institute of Ophthalmology, University College London, London, UK; UCL Genetics Institute, University College London, London, UK; Moorfields Eye Hospital, London, UK; Department of Medical Genetics, University of Cambridge, Cambridge, UK; Department of Ophthalmology UCSF Medical School, San Francisco, USA; Centre for Ophthalmology and Visual Science, Lions Eye Institute, University of Western Australia, Perth, Australia; Department of Ophthalmology, Royal Perth Hospital, Perth, Australia; Department of Ophthamology, Cambridge University Hospitals NHS Foundation Trust, Cambridge, UK

## Abstract

**Background:**

*DMBT1* is a gene that shows extensive copy number variation (CNV) that alters the number of bacteria-binding domains in the protein and has been shown to activate the complement pathway. It lies next to the *ARMS2*/*HTRA1* genes in a region of chromosome 10q26, where single nucleotide variants have been strongly associated with age-related macular degeneration (AMD), the commonest cause of blindness in Western populations. Complement activation is thought to be a key factor in the pathogenesis of this condition. We sought to investigate whether DMBT1 CNV plays any role in the susceptibility to AMD.

**Methods:**

We analysed long-range linkage disequilibrium of *DMBT1* CNV1 and CNV2 with flanking single nucleotide polymorphisms (SNPs) using our previously published CNV and HapMap Phase 3 SNP data in the CEPH Europeans from Utah (CEU). We then typed a large cohort of 860 AMD patients and 419 examined age-matched controls for copy number at *DMBT1* CNV1 and CNV2 and combined these data with copy numbers from a further 480 unexamined controls.

**Results:**

We found weak linkage disequilibrium between *DMBT1* CNV1 and CNV2 with the SNPs rs1474526 and rs714816 in the *HTRA1*/*ARMS2* region. By directly analysing copy number variation, we found no evidence of association of CNV1 or CNV2 with AMD.

**Conclusions:**

We have shown that copy number variation at *DMBT1* does not affect risk of developing age-related macular degeneration and can therefore be ruled out from future studies investigating the association of structural variation at 10q26 with AMD.

## Background

Age-related macular degeneration (AMD) is the leading cause of severe visual impairment in individuals over the age of 50, and affects the central region of the retina (the macula) containing the highest concentration of cone photoreceptors responsible for normal visual acuity [1–3]. Although the etiology and pathogenesis of AMD are not fully understood, numerous studies indicate that risk factors are both genetic and environmental include age, sex, ethnicity, smoking, hypertension and diet [4, 5]. Despite the multifactorial nature of AMD, and variable phenotype definitions, two genetic regions at 1q32 and 10q26 have been repeatedly implicated by linkage analysis and subsequently by genome-wide association studies [5–13]. The estimated effect sizes of the index SNPs at these two loci are also notable, with a fifty-fold increase in AMD risk in those individuals who are homozygous at both loci, with 65 % of AMD cases attributable to variation at these two SNPs [14].

The genetic signal at 1q32 has subsequently been shown to be due to variation involving genes of the Regulators of Complement Activation (RCA) alpha block, including complement factor H (*CFH*) and the complement factor H related genes (*CHFR1-5*). The Y402H polymorphism in CFH is strongly associated with AMD as well as several other SNPs in the *CFH* region [5, 15, 16]. Furthermore, an 84 kb deletion removing the *CFHR3* and *CHFR1* genes shows protection against AMD [17, 18]. This deletion is part of a spectrum of different copy number variants within the RCA region, with deletion and duplication mediated by different segmental duplications, and there is suggestive evidence that a rare deletion involving *CFHR1* and *CFHR4* is associated with bilateral geographic atrophy, one of the two main phenotypic variants of AMD [19, 20]. In addition to the identification of a strong genetic association between AMD and the RCA region, variation within several other complement genes such as complement factor B (CFB)/complement 2 (*C2*) [21], complement 3 (*C3*) [22] and complement factor I (*CFI*) [23] has been found to be associated with AMD. Taken together, these findings point to an important role of the complement innate immune response in the etiology of AMD [24].

The functional basis of the association at 10q26 remains unclear. Although there is some evidence that rs10490924 in the *ARMS2* gene affects systemic complement activation [25], this remains controversial and the genes *HTRA1* and *PLEKHA1* do not have convincing links with the complement pathway. In contrast, the *DMBT1* (Deleted in Malignant Brain Tumors 1) gene, 106 kb distal of rs10490924, encodes a glycoprotein which is known to bind complement C1Q [26] activate complement by the mannose-binding lectin pathway [27, 28], and promotes VEGF expression. *DMBT1* (also known as gp340, salivary agglutinin, muclin or hensin) is present on the surfaces of the eye, being abundant in tears and in the lacrimal glands with a lesser amount detected in cornea and conjunctiva [29], and is expressed in the retina [30].

Sequence variation within and surrounding *DMBT1* is poorly represented on SNP genotyping chips. However, given the distance between the rs10490924 risk allele and *DMBT1*, it is unlikely that a common single nucleotide polymorphism around *DMBT1* is responsible for the association with AMD. We considered it possible, however, that the association may, at least in part, be due to a synthetic association with a copy number variant (CNV) of very strong effect size. This might be possible particularly if that CNV was rare or due to recurrent mutation that happened to occur on a rs10490924 risk allele background, and therefore be in LD with that allele [31]. Indeed, the observation that 10q26 had been identified in linkage studies of AMD is not inconsistent with a synthetic association with a rare or moderate frequency allele of very strong effect.

**Fig. 1:**
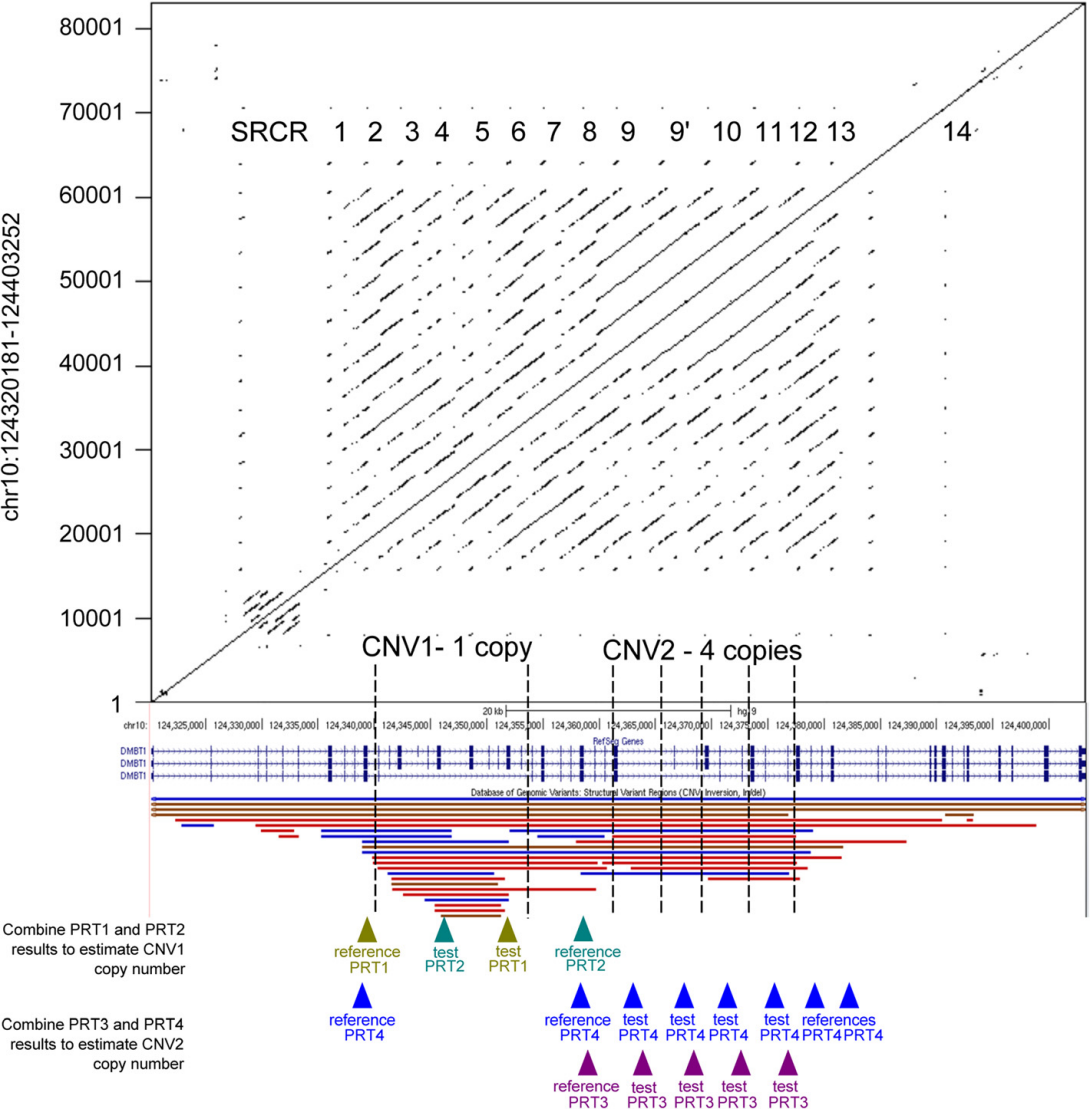
Overview of copy number variation within the *DMBT1* gene. The top half shows a dot plot of the *DMBT1* gene (shown below, in blue, from a screenshot from the UCSC genome browser) aligned against itself. Black dots indicate regions of sequence identity, with the diagonal lines showing the tandemly-repeated nature of the *DMBT1* gene. The tandemly-arranged SRCR repeat regions are annotated as numbers on this dot plot, including SRCR14 which does not bind bacteria [44]. The bottom half shows the genome assembly of the *DMBT1* gene, with one assembled copy of CNV1 and four assembled copies of CNV2. CNV regions, as recorded in the Database of Genomic Variants, are shown below the *DMBT1* gene structure. Below these, location of reference and test amplicons of the four independent paralogue ratio tests (PRTs) that measure copy number of CNV1 and CNV2 are shown

We and others have previously shown *DMBT1* exhibits extensive copy number variation that affects the number of scavenger-receptor cysteine-rich domains (SRCR) within the protein [32–34] (Fig. 1). The copy number variation is confined to two loci within the gene, termed CNV1 and CNV2. Both show a high copy number mutation rate (of the order of 1–2 % per generation) and copy number at the two loci is not correlated at the population level. There is extensive variation at the population level, such that individuals are predicted to have between 7 and 20 SRCR repeats per *DMBT1* molecule.

Investigating the relationship between flanking SNP variation and CNV also casts light on the extent to which CNV can be tagged by flanking SNPs because of linkage disequilibrium (LD). Even partial tagging would allow CNV to be indirectly imputed rather than directly genotyped, and this remains a topic of considerable interest in the literature. This is primarily because even imperfect imputation could indicate a role in disease susceptibility for certain CNVs, particularly given extremely large sample sizes. It is generally established that, using current imputation panels, whether a CNV is taggable depends on its mutational history, with CNVs generated by a unique mutational event such as most simple deletions and duplications being more taggable than complex multiallelic CNVs generated by recurrent mutation [35, 36].

We sought to explore the role of *DMBT1* CNV in AMD by investigating the linkage disequilibrium between copy number variations at *DMBT1* and common SNP alleles surrounding the gene and the association of *DMBT1* CNV with AMD in a large case–control cohort.

## Methods

### AMD case–control collection, DMBT1 CNV and SNP genotype data

The AMD case–control collection comprised cases with predominantly advanced AMD (either geographic atrophy or choroidal neovascularization) and spouse controls recruited from hospital ophthalmic clinics in London and the South East of England [22]. All subjects were examined by an ophthalmologist and had colour, stereoscopic fundus photography of the macular region. All the images were graded at the Reading Centre, Moorfields Eye Hospital, London using the International Classification of Age-related Maculopathy and Macular Degeneration [37]. All subjects described themselves as “white” on a recruitment questionnaire. DNA samples were obtained from a total of 1533 individuals. Each DNA plate contained both patients and controls and those undertaking the analysis were unaware of disease status of each sample.

*DMBT1* CNV data from the HapMap Phase 1 CEU cohort and the UK Human Random Control (HRC) cohort was published previously [34]. SNP genotype data for the Phase 3 (release 27) HapMap 1 CEU cohort was downloaded from the International HapMap Project (ftp://ftp.ncbi.nlm.nih.gov/hapmap/). SNP genotype data for the AMD case–control cohort was available from previous whole-genome SNP genotyping studies [5].

### DMBT1 copy number typing

Typing of diploid copy number of DNA samples used paralogue ratio test methods previously described [34], which involves amplification of 10 ng of DNA using fluorescently labelled primers matching both test and reference loci. Briefly, CNV1 was typed using two distinct PRT assays, PRT1 and PRT2, with CNV2 typed using a further two distinct PRT assays, PRT3 and PRT4. Copy numbers for CNV1 and CNV2 were subsequently called using a Gaussian mixture model on the mean value of both PRTs, implemented using the CNVtools v1.42.3 package for the statistical software R v2.15.3 [38]. Previous analysis has shown that these assays have an error rate of 0.37 % for CNV1 and 0.33 % for CNV2 [34].

### Linkage disequilibrium and association analysis

Linkage disequilibrium (LD) in HapMap trio data was analysed using Haploview [39]. Association analysis between CNV and flanking SNPs on HapMap CEU founder individuals was performed using PLINK v.1.0.7, using an additive model for the SNPs, treating CNV copy number as a quantitative trait, and visualised using LocusZoom [40]. Samples from the AMD dataset where *DMBT1* CNV was called were matched to clinical data and case–control association analysis was carried out on late AMD cases and examined controls using logistic regression and Stata (version 13.1, StataCorp LP, College Station, TX).

## Results and discussion

We began this study by using our previously published *DMBT1* CNV data [34] and publically available SNP data on the HapMap CEU population, as a representative of a north-west European population, to investigate any evidence for long-range LD involving *DMBT1* CNV1 and CNV2. There are several problems inherent in examining the LD between multiallelic copy number variants and SNPs. Firstly, multiallelic CNVs are often within regions of segmental duplication, where SNPs cannot be genotyped, and indeed may not exist as true diallelic polymorphisms. Secondly, most methods that measure copy number variation, such as qPCR, PRT, arrayCGH and sequence read-depth, rely on dosage information. This dosage information is for both alleles of the CNV, so that the resulting dosage is a sum of the two alleles at a diploid locus. Resolving this dose information into a true copy number genotype – for example determining whether a diploid copy number of 4 is a 2–2 genotype, 3–1 genotype or 4–0 genotype – requires observation of copy number in extended pedigrees so that the constituent alleles segregate as different combinations in different offspring.

**Fig. 2:**
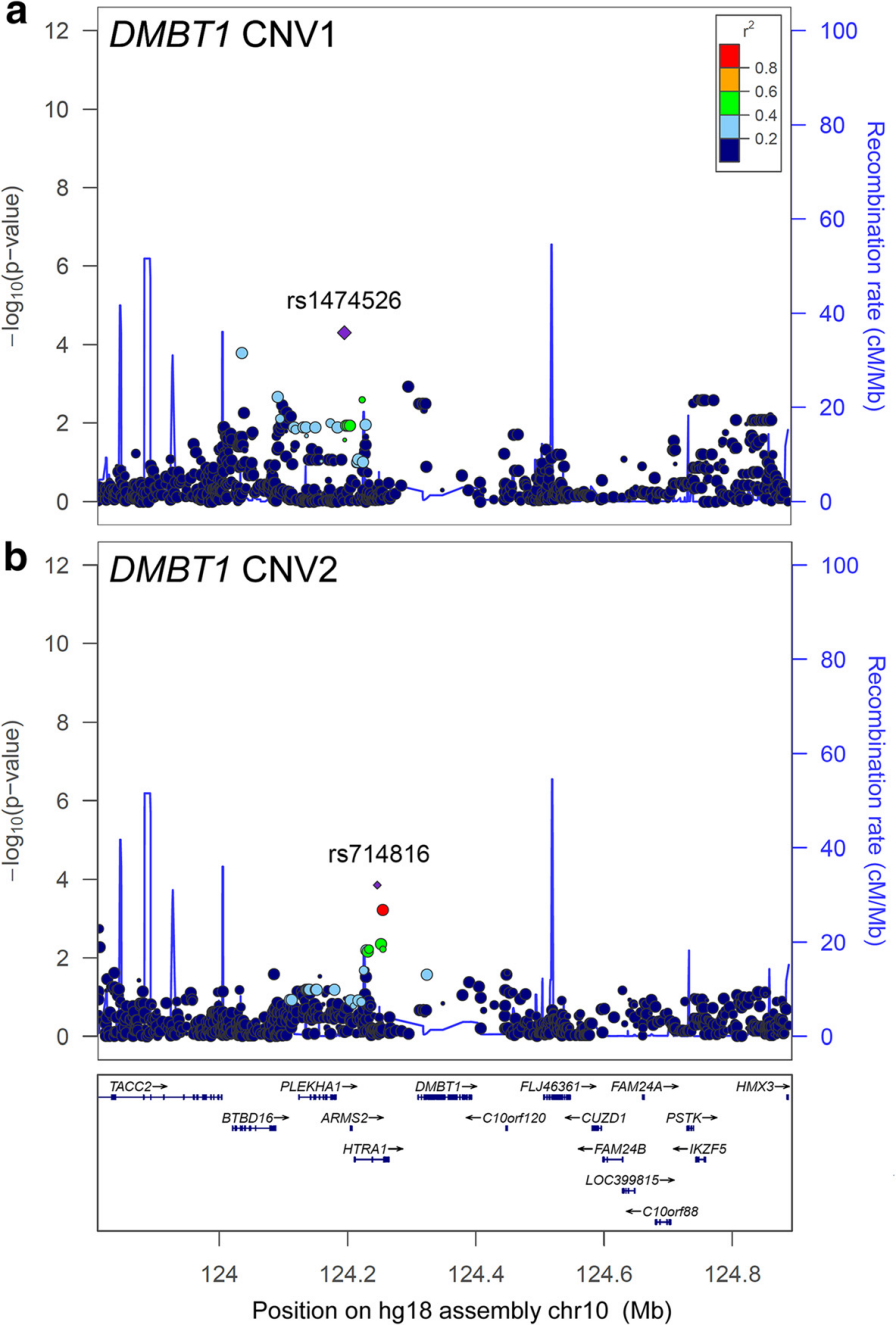
Regional association plots of *DMBT1* copy number variation and flanking SNPs. Analysis of SNPs within the 1 Mb region flanking *DMBT1* and association with diploid copy number of **a** CNV1 and **b** CNV2. The left-hand y-axis shows statistical support for an association, as measured by the negative log10 of the p-value of the linear regression test of association. The right-hand y-axis shows the recombination rate inferred from HapMap population data. The index SNP is shown as a diamond, and is annotated with its rs number. The size of each point reflects the number of genotypes for that particular SNP, and the colour of each point reflects the strength of association with the index SNP

Although the HapMap SNP data provides a densely SNP genotyped map of the human genome, the SNP density is low within and surrounding the *DMBT1* gene (Fig. 2), such that only long-range LD can be examined. For CNV1 of *DMBT1*, we directly determined diploid genotypes of HapMap CEU trios using long PCR. Because most CNV1 variation can be regarded as presence or absence of a deletion allele, we recoded this variation as diallelic, equivalent to the *DMBT1*^*SR47*-^ deletion reported previously [32], such that a copy number of 0 is regarded as a homozygous deletion, a copy number of 1 as a heterozygous deletion and copy numbers of 2 and higher as homozygous non-deleted. This allowed conventional determination of linkage disequilibrium. Pairwise LD of CNV1 with rs10490924, which has previously been shown to be most strongly associated with AMD, showed a D’ value of 1 and r^2^ value of 0.031, although the logarithm of odds (LOD) score, was only 0.75, reflecting weak confidence in the value of D’.

We also treated copy number for the two independent CNVs within *DMBT1* as a quantitative trait for association analysis in the unrelated CEU individuals. The lack of genotype information for CNVs is likely to result in a loss of power. Furthermore, an r^2^ value, reflecting the squared correlation coefficient of the SNP genotype and copy number, is used as a measure of allelic association, but is not directly comparable to r^2^ values between SNPs. Nevertheless, such information gives an indication of the relationship between a CNV and flanking SNPs. In general, as expected, there is weak association between copy number and flanking SNPs. Copy number at CNV1 is associated with rs1474526 (r^2^ = 0.14, *p* = 3.2 × 10^−3^), a *PLEKHA1*/*ARMS2* intergenic SNP which itself is only in weak LD with flanking SNPs (Fig. 2a). For CNV2, strongest association was with rs714816 (r^2^ = 0.12, *p* = 6.6 × 10^−3^, Fig. 2b), which is within the intron of the *HTRA1* gene and has been previously associated with AMD, although it is not the most strongly associated in the region [41].

**Fig. 3:**
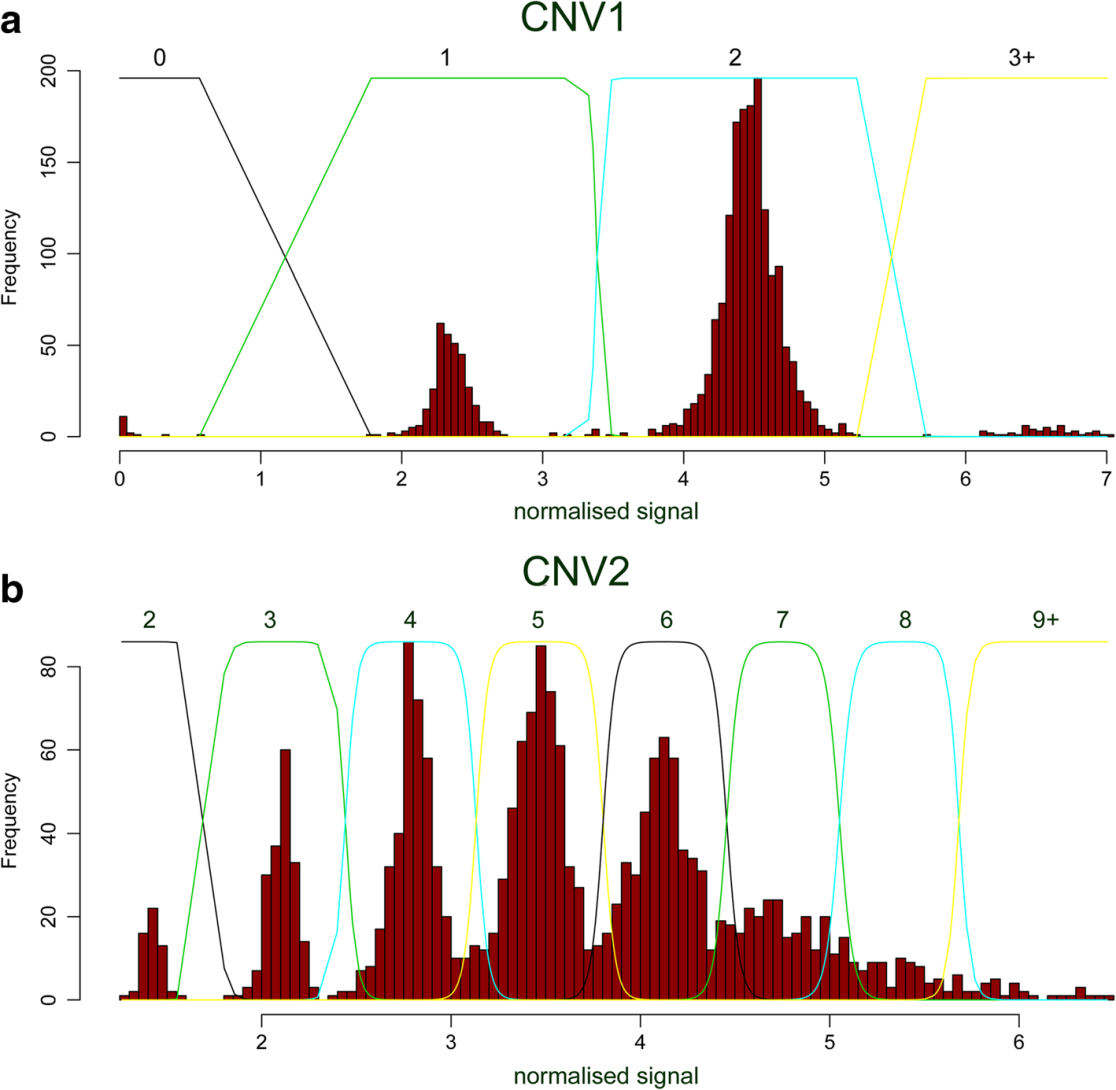
Raw copy number histograms and Gaussian mixture models. Raw copy number data, plotted as a histogram, for **a** CNV1 and **b** CNV2. The normalised signal, shown on the x-axis, reflects raw average PRT values normalised so that the overall standard deviation of the data is 1. Lines indicate the Gaussian mixture model fit, with numbers above each curve indicating the diploid copy number inferred

Given the suggestive evidence at least some allelic association between CNVs and SNPs around the *ARMS2* and *HTRA1* genes, as well as the strong functional candidacy of *DMBT1*, we directly tested for an association of CNV1 or CNV2 copy number with AMD using a case–control design. Copy number typing of cases and controls showed raw values that clustered about integer copy numbers, and integer copy number was assigned to each individual using Gaussian mixture modelling (Fig. 3). Of 1533 DNA samples of cases and examined controls typed for this study, 5 had a posterior probability of a call less than 0.99 for CNV1, of which 2 were excluded following retesting, and for CNV2, 328 out of 1533 had a had a posterior probability of a call less than 0.99 but all had a posterior probability >0.5 for any given call. A small number of outliers (for example one DNA sample with CNV1 copy number 4 sample) were removed prior to Gaussian mixture model calling of copy number, and copy number called manually. The samples where copy number was called were matched to clinical data and the resulting 860 advanced AMD cases and 419 examined controls were used in the case–control association analysis. Copy number distributions ranged from 0 to 4 for CNV1, and from 2 to 13 for CNV2, consistent with previous studies (Tables 1, 2, [34]). We found no association of diploid copy number with disease status (Table 3). We also reclassified CNV1 as *DMBT1*^*SR47*-^ deletion as previously [34], and found no association of CNV1 or CNV2 with disease status, either using matched examined controls or larger control cohort including 479 unexamined controls from the HRC cohort (Table 3). Furthermore, because the AMD cases and examined controls had been genotyped for rs10490924, we were able to analyse LD between this SNP and *DMBT1*^*SR47*-^. There was no evidence of LD between the two loci (r^2^ = 0.004, D’ = 0.25). As expected, rs10490924 genotype was strongly associated with AMD (Table 4).

**Table 1:** Frequency of CNV1 in advanced AMD cases and controls

CNV1	Cases		Controls (matched)		Controls (HRC^a^)	
	Count	Frequency	Count	Frequency	Count	Frequency
0	8	<0.01	6	0.01	2	<0.01
1	141	0.16	75	0.18	79	0.16
2	687	0.80	329	0.79	387	0.81
3	23	0.03	8	0.02	11	0.02
4	1	<0.01	0	0	1	<0.01
total	860		418		480	

^a^Human Random Control collection

**Table 2:** Frequency of CNV2 in advanced AMD cases and controls

CNV2	Cases		Controls (matched)		Controls (HRC)	
	Count	Frequency	Count	Frequency	Count	Frequency
2	27	0.03	11	0.03	11	0.02
3	83	0.10	45	0.11	48	0.1
4	162	0.19	79	0.19	110	0.23
5	230	0.27	114	0.27	125	0.26
6	206	0.24	104	0.25	104	0.22
7	84	0.10	46	0.11	51	0.11
8	44	0.05	13	0.03	23	0.05
9	18	0.02	7	0.02	4	<0.01
10	4	<0.01	0	0	4	<0.01
11	1	<0.01	0	0	0	0
12	0	0	0	0	0	0
13	1	<0.01	0	0	0	0
total	860		419		480

**Table 3:** Association of *DMBT1* CNV1 and CNV2 copy number with advanced AMD

Variation	Control group	Odds ratio (95 % CI)	*p*-value
CNV1	Matched controls (unadjusted)	1.18 (0.92–1.51)	0.20
	Matched controls (adjusted for age, sex and smoking status)	1.15 (0.88–1.51)	0.30
	All Controls	1.06 (0.86–1.30)	0.57
CNV2	Matched controls	1.04 (0.96–1.12)	0.32
	Matched controls (adjusted for age, sex and smoking status)	1.01 (0.93–1.10)	0.81
	All Controls	1.03 (0.98–1.11)	0.22
*DMBT1* ^*SR47*-^	Matched controls	0.87 (0.66–1.14)	0.31
	Matched controls (adjusted for age, sex and smoking status)	0.89 (0.66–1.19)	0.43
	All controls	0.96 (0.77–1.21)	0.73

**Table 4:** Association of rs10490924 with advanced AMD

	Cases		Controls	
	Count	Frequency	Count	Frequency
GG	250	0.29	260	0.62
GT	423	0.50	140	0.34
TT	175	0.21	16	0.04
Total	848	1.00	416	1.00
missing genotypes	12	-	3	-

Unadjusted *p* value 7.8 × 10^−30^ OR = 3.25 (95 % CI: 2.65–3.98), *n* = 1264

Adjusted p value (for age, sex, smoking) 1.5 × 10^−28^ OR = 3.37 (95 % CI: 2.72–4.18), *n* = 1175

Sequence variation within and immediately surrounding the *DMBT1* gene was not investigated for association with AMD in this study. It is poorly represented on genotyping chips, and uncertainties in aligning short sequence reads to a tandemly-repeated structure have prevented a full interpretation of variation of the gene. Nevertheless, analysis of low-coverage sequencing data outside the known CNV regions has led to the 5′ end of *DMBT1* being identified as a region that has been subject to balancing selection [42]. The role of a rapidly-mutating CNV, putatively under selection pressure, on surrounding SNP variation remains to be explored. However, although such SNP variation may be effectively untagged by current SNP markers, it is very unlikely to account for the strong association signal with AMD at 10q26 because of the clear breakdown of LD between SNPs from rs10490924 towards *DMBT1*.

Could variation at rs10490924 be affecting expression levels of *DMBT1*? Initial examination of eQTL datasets suggests not, although such long range interactions are very likely; indeed, three SNPs within *DMBT1* are eQTLs for *HTRA1* in monocytes [43]. It should also be noted that no results from an eQTL analysis of retinal tissues, or indeed any tissues where *DMBT1* is significantly expressed, have been published.

## Conclusion

We have shown that copy number variation at *DMBT1* does not affect risk of developing age-related macular degeneration, and can therefore be ruled out from future studies investigating the nature of association signal at 10q26.
